# Genome-Wide Association Study Reveals the Genetic Basis of Total Flavonoid Content in Brown Rice

**DOI:** 10.3390/genes14091684

**Published:** 2023-08-25

**Authors:** Haijian Xia, Xiaoying Pu, Xiaoyang Zhu, Xiaomeng Yang, Haifeng Guo, Henan Diao, Quan Zhang, Yulong Wang, Xingming Sun, Hongliang Zhang, Zhanying Zhang, Yawen Zeng, Zichao Li

**Affiliations:** 1 Beijing Key Laboratory of Crop Genetic Improvement, College of Agronomy and Biotechnology, China Agricultural University, Beijing 100193, China; hjxiaynyx@126.com (H.X.);; 2 Biotechnology and Germplasm Resources Institute, Yunnan Academy of Agricultural Sciences/Agricultural Biotechnology Key Laboratory of Yunnan Province, Kunming 650205, China; puxiaoying@163.com (X.P.);; 3 Heihe Branch of Heilongjiang Academy of Agricultural Sciences, Heihe 164300, China; 4 Sanya Institute, China Agricultural University, Sanya 572025, China

**Keywords:** flavonoid, functional rice, gene, germplasm resource, QTL

## Abstract

Flavonoids have anti-inflammatory, antioxidative, and anticarcinogenic effects. Breeding rice varieties rich in flavonoids can prevent chronic diseases such as cancer and cardio-cerebrovascular diseases. However, most of the genes reported are known to regulate flavonoid content in leaves or seedlings. To further elucidate the genetic basis of flavonoid content in rice grains and identify germplasm rich in flavonoids in grains, a set of rice core collections containing 633 accessions from 32 countries was used to determine total flavonoid content (TFC) in brown rice. We identified ten excellent germplasms with TFC exceeding 300 mg/100 g. Using a compressed mixed linear model, a total of 53 quantitative trait loci (QTLs) were detected through a genome-wide association study (GWAS). By combining linkage disequilibrium (LD) analysis, location of significant single nucleotide polymorphisms (SNPs), gene expression, and haplotype analysis, eight candidate genes were identified from two important QTLs (*qTFC1-6* and *qTFC9-7*), among which *LOC_Os01g59440* and *LOC_Os09g24260* are the most likely candidate genes. We also analyzed the geographic distribution and breeding utilization of favorable haplotypes of the two genes. Our findings provide insights into the genetic basis of TFC in brown rice and could facilitate the breeding of flavonoid-rich varieties, which may be a prevention and adjuvant treatment for cancer and cardio-cerebrovascular diseases.

## 1. Introduction

An increasing number of people are in a sub-health state and suffer from various chronic diseases, such as cancer and cardio-cerebrovascular disease [1,2,3]. Ten million people in the world will have died of cancer in 2020 [4]. Hypertension is the main risk factor for cardio-cerebrovascular disease [5]. Globally, more than a quarter of the population is hypertensive [6]. Therefore, reducing the harm that these diseases pose to human health is essential.

Flavonoids are important secondary metabolites widely found in plants [7] and can be classified into six major subgroups: chalcones, flavones, flavonols, flavandiols, anthocyanins, and proanthocyanidins [8]. Flavonoids have anti-inflammatory, antioxidative, and anticarcinogenic properties useful for the prevention and adjuvant treatment of cancer and cardio-cerebrovascular disease [9]. Rice is the main staple and energy source for more than half of the world’s population [10]. Breeding functional rice varieties rich in flavonoids could increase daily flavonoid intake through diet without the need for drugs, which may be an easy and convenient way to prevent cancer and cardio-cerebrovascular diseases. To achieve this goal, it is essential to identify rice germplasm with high flavonoid content and understand the genetic basis of flavonoid content in rice grains.

Previous studies identifying rice germplasm rich in flavonoids are relatively rare. Li et al. (2022) determined the total flavonoid content (TFC) of 164 accessions from the United States Department of Agriculture’s (USDA) rice mini-core collection. The TFC of 905 accessions from the primary core collection in Yunnan province, China, was also identified [11]. In addition, the TFC of less than 20 Indian and South Korean rice varieties was identified [12,13,14,15]. Therefore, to identify germplasm rich in flavonoids, it is necessary to select more varieties with a wider geographical distribution and greater genetic variation.

Several studies have been conducted to elucidate the genetic basis of the flavonoid content of rice. The genes affecting flavonoid content in rice can be divided into four categories: structural, regulatory, transporter, and modifying enzyme genes [16]. Structural genes encode proteins that catalyze the conversion of phenylalanine into chalcones, flavones, flavonols, flavandiols, anthocyanins, and proanthocyanidins, such as *OsPAL06* [17], *OsF3H* [18], *OsFLS* [19], and *OsANS* [20]. Regulatory genes encode proteins that regulate the expression of structural genes, thereby affecting flavonoid content, such as *Rc* [21], *OsB1* [22], and *OsC1-MYB* [20]. The modifying enzymes catalyze the glycosylation or methylation of flavonoids to increase their stability, such as *OsUGT706C2* [23] and *OsNOMT* [24]. The transporters can transfer anthocyanins from the cytoplasm to vacuoles, such as *OsMATE34* [25].

Although several genes that affect flavonoid content in rice have been cloned, most of the reported genes are known to influence flavonoid content in leaves or seedlings [22,26,27,28,29]. To our knowledge, only 11 genes affecting flavonoid content in grains have been reported, including three structural genes (*DFR* [20], *OsF3’H* [18], and *CYP75B4* [30]) and eight regulatory genes. Dihydroflavonol 4-reductase (DFR) catalyzes the conversion of dihydroflavonol to flavonol in rice pericarps. OsF3’H catalyzes the formation of eriodictyol in the seed coat. CYP75B4 hydroxylates leucoanthocyanins to produce anthocyanins and proanthocyanidins. Among the eight regulatory genes, five encode transcription factors (*OsMYB3* [31], *OsB2* [22], *Rc* [21], *OsTTG1* [32], and *OsVP1* [33]), and three encode non-transcription factor proteins (*OsDET1*, *OsDDB1*, and *OsCOP10* [34]). Rc, OsMYB3, OsB2, OsTTG1, and OsVP1 regulate the expression of some structural genes to affect flavonoid content in rice pericarps. OsDET1, OsDDB1, and OsCOP10 combine to form a complex device driver (CDD), which affects the flavonoid biosynthesis pathway in rice seeds.

To further elucidate the genetic basis of TFC in rice grains and identify flavonoid-rich varieties, we used a rice core collection population of 633 varieties to determine TFC in brown rice. We identified ten excellent germplasms with high TFC. A total of 53 (quantitative trait loci) QTLs were detected through a genome-wide association study (GWAS). Through linkage disequilibrium (LD) analysis, gene expression, and other methods, we identified eight candidate genes from two preferred QTLs. Our study sheds further light on the genetic basis of TFC in brown rice and could facilitate the breeding of flavonoid-rich varieties.

## 2. Materials and Methods

### 2.1. Plant Materials

**Figure 1:**
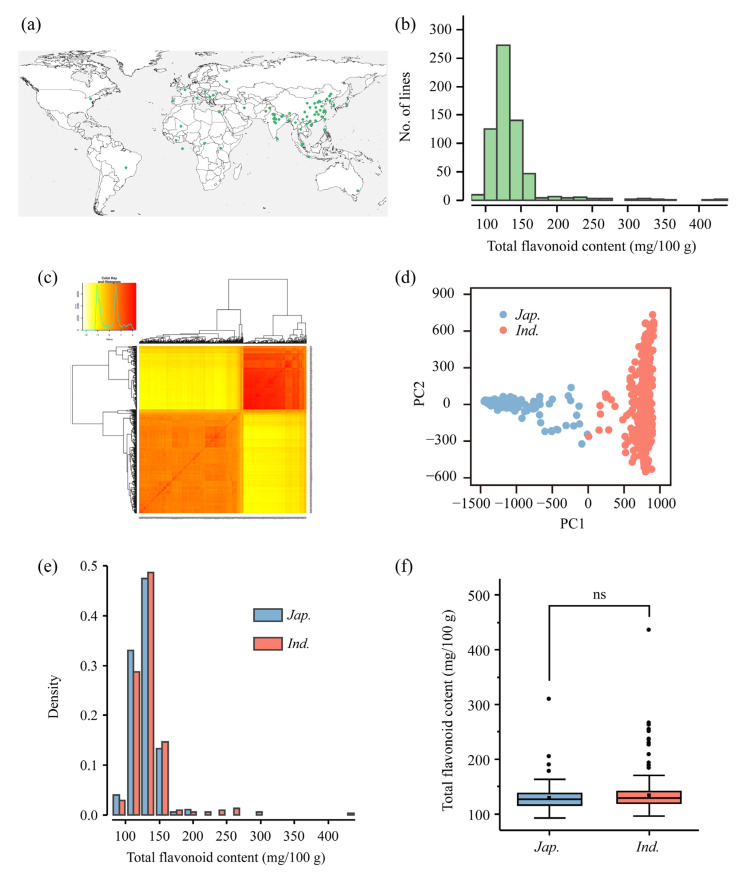
Phenotypic diversity of total flavonoid content (TFC) and population structure of 633 cultivated rice. (**a**) Geographic distribution of all accessions. Each green dot represents a accession. (**b**) A histogram showing the distribution of TFC in the full population. (**c**) Kinship plot of all accessions. (**d**) Principal component analysis plot. (**e**) Histogram of TFC in the *indica* and *japonica* subpopulations. The *Y*-axis shows the proportion of accessions falling within a certain TFC range relative to the total number of accessions within each subpopulation. (**f**) Boxplots showing the phenotypic variation of TFC in the *indica* and *japonica* subpopulations.

This study used 633 cultivated rice accessions, comprising 237 accessions from the rice mini-core collection [35] and 396 accessions from the International Rice Molecular Breeding Network [36]. These accessions were collected from 32 countries across five continents (583 from Asia, 18 from Africa, 15 from America, 9 from Europe, and 8 from Oceania) (Figure 1a), representing a wide range of genetic diversity. Detailed information on the accessions is provided in Table S1.

### 2.2. Field Trials and Measurement of Total Flavonoid Content

Seeds from the 633 rice accessions were planted in Yuxi, Yunnan, China, in 2015, with two replications. A randomized complete block design was used. After harvesting and drying, 20 g of dehulled brown rice from each accession were ground to determine the TFC.

The TFC was determined using the NaNO_2_-Al(NO_3_)_3_ method [37]. In a 16 mL centrifuge tube, a powdered rice sample (0.5 g) was collected and mixed with 5 mL of 50% ethanol. The mixture was oscillated in a vibrator (manufacturer: Jiangsu Jieruier Electric Appliance Co., Ltd., Changzhou, China; model: SHA-B) at room temperature and 200 rpm for a duration of 3 h, then centrifuged at 7000 rpm for 3 min. After centrifugation, 1 mL of supernatant was placed in a new tube, mixed with 0.6 mL of 5% NaNO_2_, and allowed to stand for 5 min. The same procedure was performed with 0.6 mL of 10% Al(NO_3_)_3_ and 4 mL of 5% NaOH, and the tubes were allowed to stand for 6 min and 10 min, respectively. After the color became red and stabilized, the absorption was measured at a wavelength of 500 nm. A standard curve was developed using different concentrations (0.05, 0.1, 0.15, 0.2, and 0.25 mg/mL) of rutin standard obtained from Guizhou Dida Science and Technology Limited Company (Guiyang, China). The TFC was estimated using the equations *y* = 1.8764*x* − 0.0054 (*R*^2^ = 0.9997) and *y* = 1.8503*x* − 0.0129 (*R*^2^ = 0.9995) for the two replications, respectively. The regression equations and coefficients of determination indicated a high level of precision for the measurement of TFC. To reduce error, all analyses were repeated three times. The TFC was expressed as milligrams of rutin equivalent per 100 g of dry rice flour.

### 2.3. Genotype

The raw SNP genotype data for the 633 accessions were obtained from the 3000 Rice Genome Project (3KRGP) [38,39] with an average sequencing depth of 15×. SNPs with more than two alleles, a missing rate over 30%, and a minor allele frequency (MAF) less than 5% were removed, resulting in 2762746 SNPs that were used in population structure analysis and GWAS.

### 2.4. Population Structure Analysis

Using PLINK version 1.9 (window 50 bp, step size 5 bp, *r*^2^ < 0.3) [40], 276464 SNPs in linkage equilibrium were screened and used to perform principal component analysis using Genome-wide complex trait analysis (GCTA) version 1.93.2 [41]. Kinship analysis were carried out in the R programming language version 3.5.2 using the GAPIT package [42].

### 2.5. GWAS

GWAS was conducted for the full, *indica,* and *japonica* populations. A total of 2762746 high-quality SNPs (MAF ≥ 5%, missing rate < 30%) were used to perform GWAS using a compressed mixed linear model (CMLM) in the GAPIT package operated in an R environment [42]. The first three principal components and kinship were integrated simultaneously to control false positives in CMLM. The genome-wide significance threshold was determined using permutation tests with 1000 replications [43]. The significance threshold for the association analysis was set at the top 5% probability. A genome interval is defined as a QTL if it contains at least three consecutive significant SNPs and the distance between any two adjacent significant SNPs is less than 170 kb, given LD decay values of approximately 120 kb and 170 kb in *indica* and *japonica* populations, respectively [44,45]. The SNP with the minimum *p*-value within a QTL was considered the lead SNP of the QTL. If two QTLs detected in two populations had overlapping intervals, they were considered co-located QTLs.

### 2.6. Screening Candidate Genes

Screening candidate genes is a process of progressively reducing the candidate gene pool through various methods. For a selected target QTL, the LD heatmap was created using all significant SNPs in an interval of about one Mb on the flank of the lead SNP [46,47]. The region with consecutive SNPs closely linked to the lead SNP (*r*^2^ ≥ 0.6) was regarded as the local LD interval [48]. According to the gene annotation of the MSU Rice Genome Annotation Project Database and Resource (http://rice.plantbiology.msu.edu/, (accessed on 10 January 2023) [49]), all predicted genes in the LD interval were identified. Among all the predicted genes, transposons and retrotransposons were first excluded, and then genes containing significant SNPs in the entire gene region, including the promoter, exon, intron, and/or 3’UTR, were retained. For genes containing significant SNPs, their expression in grain-related tissues (ovary, embryo, and endosperm) at different time points after pollination can be examined through two rice gene expression databases, RiceXPro (https://ricexpro.dna.affrc.go.jp/ (accessed on 15 January 2023), [50]) and MSU (http://rice.uga.edu/expression.shtml (accessed on 16 January 2023), [49]). Genes with no expression in grain-related tissues are unlikely to be candidate genes, while genes with some expression in these tissues may be considered candidate genes. For candidate genes that meet the expression characteristics, haplotype analysis can be performed using significant SNPs. If the TFC of different haplotypes of a gene shows significant differences in the *japonica* or *indica* subgroups, the gene may be considered a candidate gene. If there is no significant difference in the TFC of different haplotypes of a gene in both the *japonica* and *indica* subpopulations, then this gene can be excluded.

### 2.7. Statistical Analysis and Graphing

SPSS 19 software and a two-tailed *t*-test were used to analyze the significance difference. Venn diagrams, Manhattan plots, Q-Q plots, QTL summary plots, and geographic distribution plots were drawn using R 4.2.1. Principal component analysis plots, histograms, violin diagrams, and boxplots were drawn using Origin 2022.

## 3. Results

### 3.1. Identification of Functional Rice Germplasm Rich in Flavonoids

This study utilized 633 cultivated rice accessions from 32 countries across five continents to identify rice varieties with high flavonoid content and to conduct GWAS (Figure 1a and Table S1). The TFC in brown rice from all accessions was measured using a spectrophotometer. The full population exhibited large variations in TFC (Figure 1b), ranging from 90.6 (Tieganwu, *japonica*) to 435.9 (Yanshuichi, *indica*) mg/100 g, with a mean value of 136.0 mg/100 g and a coefficient of variation (CV) of 28.2%. The TFC of Yanshuichi is 4.8 times that of Tieganwu. The distribution of TFC was positively skewed (Figure 1b), with most accessions having low TFC (ranging from 100 to 150 mg/100 g) and only a few varieties having high TFC more than 350 mg/100 g. The 10 accessions with the highest TFC were identified and listed in Table 1: Yanshuichi (435.9 mg/100 g), Feidongtangdao (426.0 mg/100 g), Lengshuigu 2 (407.3 mg/100 g), Qiuqianbai (355.8 mg/100 g), IRAT 669 (347.8 mg/100 g), Haobayong 1 (344.5 mg/100 g), Lamujia (331.5 mg/100 g), Longhuamaohulu (330.6 mg/100 g), Tianhandao (316.4 mg/100 g), and Banjiemang (313.7 mg/100 g). These elite varieties, which comprise three *indica* and seven *japonica* varieties, mostly from southern China, and 90% of which are landraces, can serve as valuable germplasm resources for breeding flavonoid-rich functional rice.

Based on the known variety information, the 633 accessions can be categorized into two subgroups: 383 *indica* and 250 *japonica* varieties. Kinship analysis and principal component (PC) analysis using the genotypes confirmed the division of all varieties into these two subgroups (Figure 1c,d). Both the *japonica* and *indica* subpopulations exhibited a positively skewed distribution of TFC (Figure 1e). Moreover, there was no significant difference in TFC between the *japonica* and *indica* subpopulations (Figure 1f).

### 3.2. GWAS for Total Flavonoid Content

**Figure 2:**
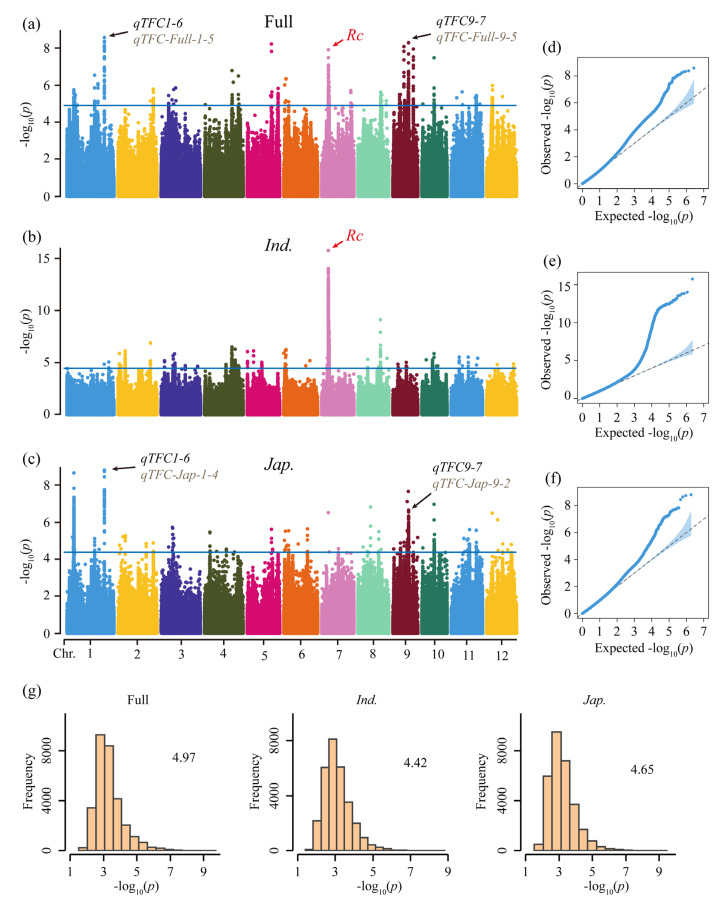
Genome-wide association study (GWAS) of TFC in brown rice in the full, *indica*, and *japonica* populations. (**a**–**c**) Manhattan plots showing the GWAS results for the full, *indica*, and *japonica* populations, respectively. (**d**–**f**) Quantile-quantile (Q-Q) plots illustrating the distribution of observed *p*-values compared to expected *p*-values in the full, *indica*, and *japonica* populations, respectively. (**g**) Histogram displaying the distribution of the maximum −log_10_ (*P*) values from 1000 permutations. The significance thresholds for GWAS are provided on the right side of each plot.

To elucidate the genetic basis of the flavonoid content in rice grains, GWAS was performed on 633 accessions. To eliminate false positives from the population structure, we performed GWAS using a compressed mixed linear model (CMLM) with PC and kinship, which accounts for population structure and identifies the optimal group kinship matrix [51]. The first three PCs were used to construct the PC matrix. The CMLM model was applied to GWAS of the full, *indica*, and *japonica* populations (Figure 2a–c). The quantile-quantile (Q-Q) plots indicated that CMLM eliminated false positives and false negatives well in the three populations (Figure 2d–f). The significant thresholds of GWAS in the three populations (full, −log_10_(*P*) = 4.97; *indica*, −log_10_(*P*) = 4.42; *japonica*, −log_10_(*P*) = 4.65) were determined using permutation tests (Figure 2g).

**Figure 3:**
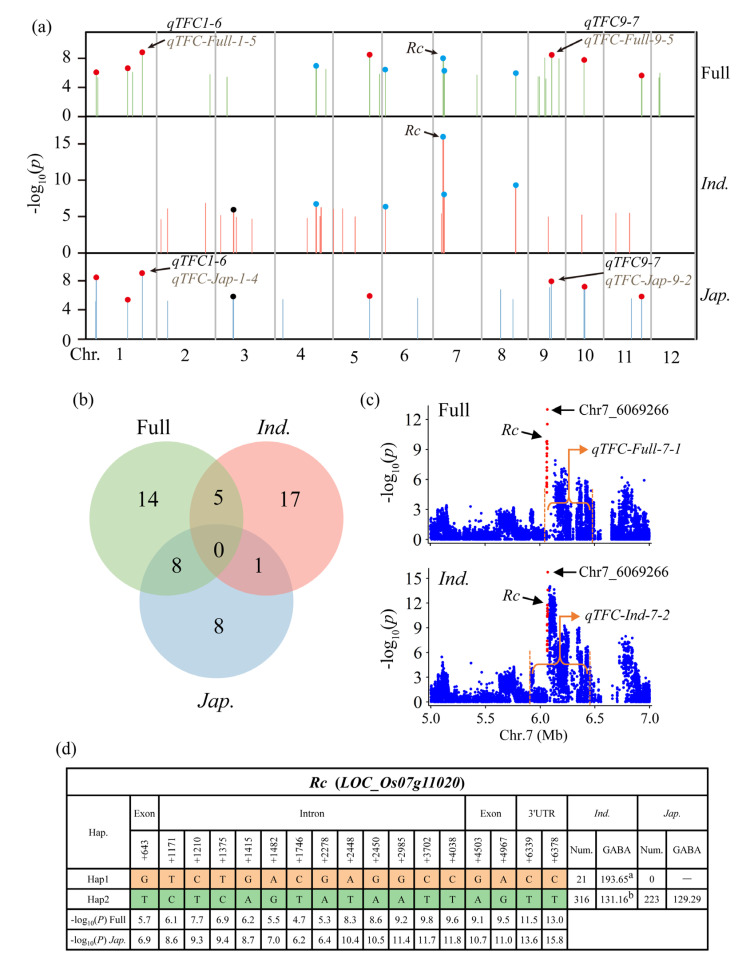
Summary of quantitative trait loci (QTLs). (**a**) Summary of QTLs in the full, *indica*, and *japonica* populations. Each bar represents a QTL. The blue dots indicate the QTLs co-located in both the full and *indica* populations. The red dots indicate the QTLs co-located in both the full and *japonica* populations. The black dots indicate the QTLs co-located in both the *indica* and *japonica* populations. (**b**) A venn diagram showing the number of QTLs identified in each population and the overlap between them. (**c**) Local Manhattan plot of chromosome 7 from 5 Mb to 7 Mb in the full population (**top**) and *indica* subpopulation (**bottom**). The interval inside the orange dotted line indicates the QTL. The red dots indicate significant SNPs located on *Rc*. Chr7_6069266 is the same lead SNP as *qTFC-Full-7-1* and *qTFC-Ind-7-2*. (**d**) Haplotype analysis of *Rc*. Different letters indicate significant differences at *p* < 0.05 according to double-tailed Student’s *t*-tests.

Ref. [51] the full population exhibited a higher number of QTLs compared to the *indica* and *japonica* subpopulations (full, 27; *indica*, 23; *japonica*, 17) (Figure 3a,b; Table S2). We identified five co-located QTLs on chromosomes 4, 6, 7, and 8 in both the full and *indica* populations, as well as seven co-located QTLs on chromosomes 1, 5, 9, 10, and 11 in both the full and *japonica* populations (Figure 3a,b; Table S2). Additionally, a co-located QTL on chromosome three was identified in both the *indica* and *japonica* subpopulations (Figure 3a,b; Table S2). However, no QTL was detected in all three populations simultaneously. Co-located QTLs are likely to contain genes that control flavonoid content in rice grains and can serve as preferred QTLs for screening candidate genes. Table 2 provides information on all co-localized QTLs. *qTFC7-2*, *qTFC1-6*, and *qTFC9-7* exhibited strong association signals with TFC in their respective populations, with more than or equal to 30 commonly significant single nucleotide polymorphisms (SNPs) and overlapping intervals greater than 25 kb. Therefore, they are the preferred QTLs for exploring candidate genes.

To verify the reliability of our results, we investigated whether the reported genes were located within candidate QTLs. Our analysis revealed that *Rc* was detected in both *qTFC-Full-7-1* within the full population and *qTFC-Ind-7-2* within the *indica* subpopulation (Figure 2a,b and Figure 3a; Table S2). *Rc* encodes a transcription factor that contains a basic helix-loop-helix (bHLH) domain, which affects proanthocyanidin synthesis by regulating the expression of some structural genes involved in flavonoid biosynthesis and is a major gene controlling flavonoid synthesis in rice pericarps [21,33,52,53]. The lead SNP of *qTFC-Full-7-1* and *qTFC-Ind-7-2* was the same SNP (Chr7_6069266) (Figure 3c), located in the 3′ untranslated region (UTR) of *Rc*. Additionally, both *qTFC-Full-7-1* and *qTFC-Ind-7-2* exhibited the strongest association signals in their respective populations (Figure 2a,b and Figure 3a; Table S2).

The GWAS results for both the full and *indica* populations identified 20 and 19 significant SNPs, respectively, located in *Rc* (Figure 3c and Table S3). All 19 significant SNPs of *Rc* in the *indica* subpopulation were also significant in the full population, including one non-synonymous SNP, two synonymous SNPs, 14 intron SNPs, and two 3′UTR SNPs (Table S3). Using these significant SNPs, all accessions were divided into two haplotypes of *Rc* (*Rc*^Hap1^ and *Rc*^Hap2^) (Figure 3d). The *indica* subpopulation contained *Rc*^Hap1^ and *Rc*^Hap2^, while all accessions in the *japonica* subpopulation belonged to *Rc*^Hap2^. The average TFC in *Rc*^Hap1^ was significantly higher than that of *Rc*^Hap2^ in the *indica* subpopulation (Figure 3d), indicating that *Rc*^Hap1^ was the favorable haplotype in the *indica* subpopulation. These findings support the notion that *Rc* is the functional gene for *qTFC-Full-7-1* and *qTFC-Ind-7-2* and further confirm that *Rc* is the major gene regulating flavonoid content. Overall, these results demonstrated that our GWAS results were reliable. Moreover, most QTLs with strong signals in both the *indica* and *japonica* subpopulations, such as *qTFC-Ind-7-2*, *qTFC-Ind-7-3*, *qTFC-Ind-8-1*, *qTFC-Jap-1-2*, *qTFC-Jap-1-3*, and *qTFC-Jap-9-2*, were also detected in the full population (Figure 3a; Table S2), providing further evidence of the reliability of our GWAS results.

### 3.3. Identification of Candidate Genes for qTFC1-6

**Figure 4:**
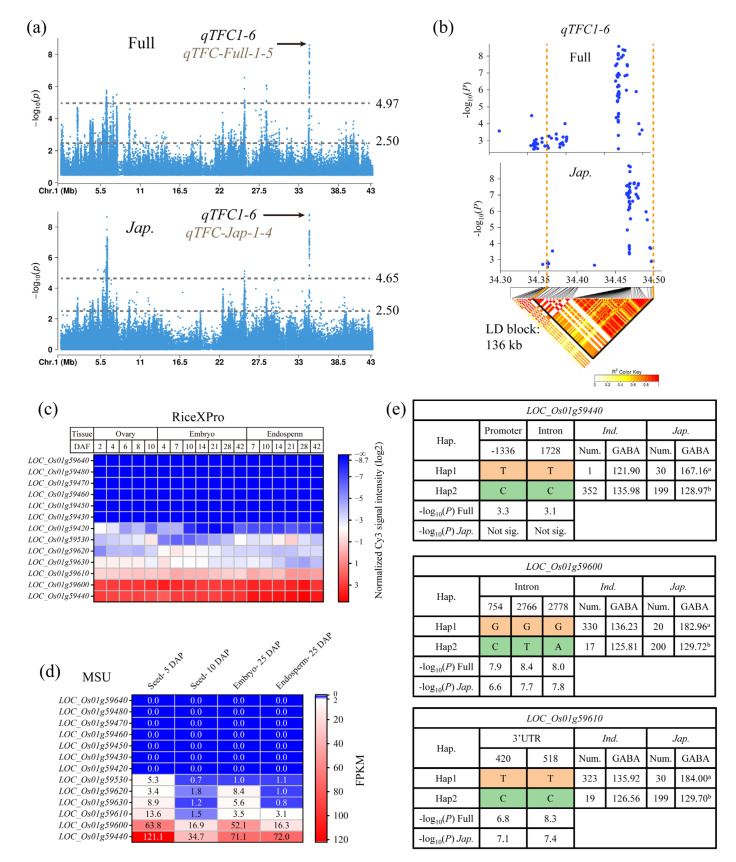
Exploration of candidate genes in *qTFC1-6*. (**a**) Manhattan plots of the full and *japonica* populations on chromosome 1. (**b**) Local Manhattan plots and linkage disequilibrium plots of *qTFC1-6* based on significant single nucleotide polymorphisms (SNPs) in the full and *japonica* populations. (**c**) Relative expression levels of the 13 primary candidate genes of *qTFC1-6* in grain-related tissues in the RiceXPro gene expression database. DAF represents the number of days after flowering. The relative expression levels are represented by the normalized Cy3 fluorescence signal intensity. Since the normalization is based on a logarithmic scale of two, genes with no expression are represented as negative infinity (−∞) in blue. (**d**) Relative expression levels of the 13 primary candidate genes of *qTFC1-6* in grain-related tissues in the MSU gene expression database. The relative expression levels are represented by fragments per kilobase of the exon model per million mapped fragments (FPKM). (**e**) Haplotype analysis of three candidate genes. Different letters indicate significant differences at *p* < 0.05 according to double-tailed Student’s *t*-tests.

*qTFC1-6* was a co-located QTL of *qTFC-Full-1-5* in the full population and *qTFC-Jap-1-4* in the *japonica* subpopulation (Table 2). Both *qTFC-Full-1-5* and *qTFC-Jap-1-4* contained clustered significant SNPs and exhibited the highest association signals in their respective populations (Figure 2a,c; Table S2). The lead SNPs for *qTFC-Full-1-5* and *qTFC-Jap-1-4* had −log_10_(*P*) values of 8.58 and 8.81, respectively (Table 2). There were 36 commonly significant SNPs between *qTFC-Full-1-5* and *qTFC-Jap-1-4*. However, due to the small interval size (only 26 kb) of *qTFC1-6*, the interval contained only six genes. From the Manhattan plot of the full population and the *japonica* subpopulation on chromosome 1, it was observed that there were still clustered SNPs belonging to these two QTLs below the threshold (Figure 4a). In addition, considering the average LD of rice is approximately 120~165 kb [44]. Therefore, to ensure the identification of reliable candidate genes, we adjusted the threshold for *qTFC-Full-1-5* and *qTFC-Jap-1-4* in their respective populations to −log_10_(*P*) = 2.50, based on the GWAS results of SNPs near these QTLs on chromosome 1 (Figure 4a). It can be seen that when the threshold was lowered to 2.50, there were not many significant SNPs near *qTFC-Full-1-5* and *qTFC-Jap-1-4* that did not belong to these two QTLs (Figure 4a), indicating that it was feasible to use the low threshold of −log_10_(*P*) = 2.50 when screening candidate genes for *qTFC1-6*. Using this new threshold, the intervals of *qTFC-Full-1-5* and *qTFC-Jap-1-4* were expanded to 186 kb (Chr1_34313030–Chr1_34499004) and 144 kb (Chr1_34355283–Chr1_34499004), respectively. Consequently, we selected the maximum interval involved in *qTFC-Full-1-5* and *qTFC-Jap-1-4* as the interval for *qTFC1-6*, which spanned from Chr1_34313030 to Chr1_34499004 and has a length of 186 kb. *qTFC1-6* contained 49 commonly significant SNPs in both the full and *japonica* populations.

Local LD analysis using all significant SNPs revealed that the LD block for *qTFC1-6* spanned from Chr1_34363101 to Chr1_34499004 (Figure 4b), covering a distance of 136 kb, and contains 22 genes, of which *LOC_Os01g59580* is a transposon that can be excluded. Among the remaining 21 genes, 13 have significant SNPs in their promoter or genebody regions. The gene responsible for regulating TFC in brown rice is likely to be expressed in grain-related tissues such as the ovary, embryo, and endosperm. To identify the promising candidate genes, we examined the expression levels of the 13 genes in these tissues at different stages after pollination using two rice gene expression databases, RiceXPro [50] and MSU [49]. It was found that *LOC_Os01g59440* and *LOC_Os01g59600* had the highest expression levels in the ovary, embryo, and endosperm at different stages after pollination, while *LOC_Os01g59610* had moderate expression, and *LOC_Os01g59630*, *LOC_Os01g59620*, *LOC_Os01g59530*, and *LOC_Os01g59420* exhibited extremely low expression levels (Figure 4c,d). *LOC_Os01g59430*, *LOC_Os01g59450*, *LOC_Os01g59460*, *LOC_Os01g59470*, *LOC_Os01g59480*, and *LOC_Os01g59640* were not expressed in these tissues at different stages after pollination (Figure 4c,d). Therefore, *LOC_Os01g59440*, *LOC_Os01g59600*, and *LOC_Os01g59610* are the most likely candidates for *qTFC1-6*. Haplotype analysis of these three genes using significant SNPs revealed that they were divided into two haplotypes (Hap1 and Hap2) in all germplasms. In the *japonica* subpopulation, the average TFC of Hap1 was significantly higher than that of Hap2, while in the *indica* subpopulation, there was no significant difference in the mean TFC between Hap1 and Hap2 (Figure 4e). This result further supports the candidacy of these three genes. Therefore, *LOC_Os01g59440*, *LOC_Os01g59600*, and *LOC_Os01g59610* were the three candidate genes for *qTFC1-6* (Table S4). *LOC_Os01g59440* encodes a receptor-like kinase. OsRLCK160 also encodes a receptor-like kinase that can interact with OsbZIP48 and phosphorylate it, thereby regulating the accumulation of flavonoids in rice leaves. Therefore, *LOC_Os01g59440* is likely to have a similar function to *OsRLCK160* and thus is more likely to be the functional gene for *qTFC1-6*; however, there is not enough evidence to exclude *LOC_Os01g59600* and *LOC_Os01g59610* from the candidates.

### 3.4. Identification of Candidate Genes for qTFC9-7

**Figure 5:**
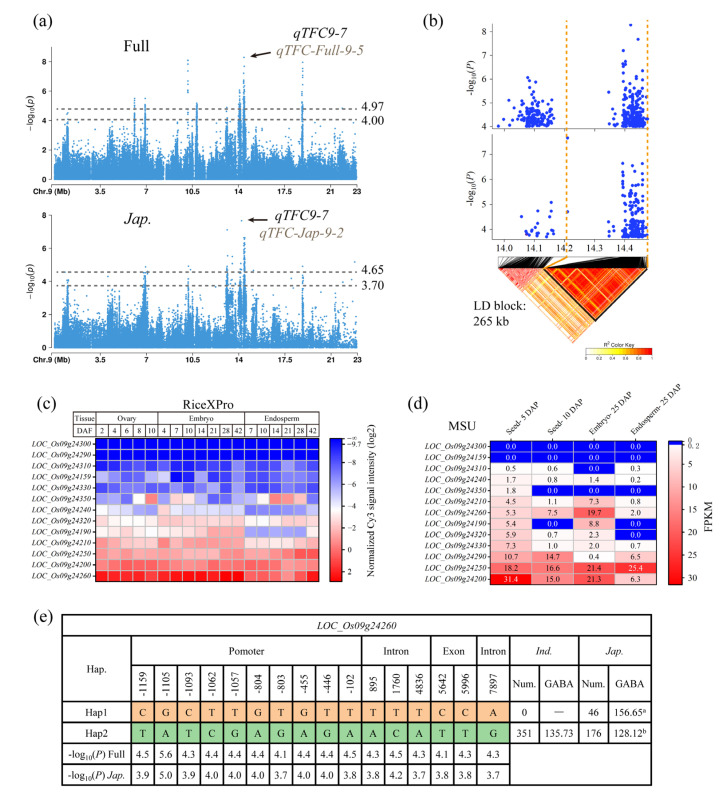
Exploration of candidate genes in *qTFC9-7*. (**a**) Manhattan plots of the full and *japonica* populations on chromosome 9. (**b**) Local Manhattan plots and linkage disequilibrium plots of *qTFC9-7* based on significant SNPs in the full and *japonica* populations. (**c**) Relative expression levels of the 13 primary candidate genes of *qTFC9-7* in grain-related tissues in the RiceXPro gene expression database. (**d**) Relative expression levels of the five primary candidate genes of *qTFC9-7* in grain-related tissues in the MSU gene expression database. (**e**) Haplotype analysis of *LOC_Os09g24260*. Different letters indicate significant differences at *p* < 0.05 according to double-tailed Student’s *t*-tests.

*qTFC9-7* was a co-located QTL of *qTFC-Full-9-5* in the full population and *qTFC-Jap-9-2* in the *japonica* subpopulation, with an overlapping interval of 78 kb (Chr9_14393345–Chr9_14459848) (Table 2). Both *qTFC-Full-9-5* and *qTFC-Jap-9-2* contained clusters of significant SNPs. *qTFC-Full-9-5* had the second highest association signal in the full population, and *qTFC-Jap-9-2* had the third highest association signal in the *japonica* subpopulation (Figure 2a,c; Table S2). The lead SNPs for *qTFC-Full-9-5* and *qTFC-Jap-9-2* had −log_10_(*P*) values of 8.29 and 7.66, respectively (Table 2). There were 30 commonly significant SNPs between *qTFC-Full-9-5* and *qTFC-Jap-9-2*. To ensure the identification of reliable candidate genes, we lowered the threshold to −log_10_(*P*) = 4.00 for *qTFC-Full-9-6* and −log_10_(*P*) = 3.70 for *qTFC-Jap-9-3* based on the GWAS results of SNPs near these two QTLs on chromosome 9 (Figure 5a). It can be seen that after the threshold was lowered, there were not many significant SNPs near *qTFC-Full-9-7* and *qTFC-Jap-9-2* that did not belong to these two QTLs (Figure 5a), indicating that it was feasible to lower the threshold when screening candidate genes for *qTFC9-7*. Based on the new threshold, the interval for *qTFC9-7* spanned from Chr9_13979678 to Chr9_14475412, covering a distance of 496 kb, and contained 79 commonly significant SNPs in both the full and *japonica* populations.

Local LD analysis using all significant SNPs showed that the LD block for *qTFC9-7* spanned from Chr9_14210097 to Chr9_14475412, covering a distance of 265 kb and containing 52 genes. After excluding transposons, retrotransposons, and hypothetical proteins, 33 genes remained, of which 13 have significant SNPs in their promoter or genebody regions. The gene responsible for regulating TFC in brown rice is likely expressed in grain-related tissues such as the ovary, embryo, and endosperm. By examining the expression levels of these 13 genes in these tissues at different stages after pollination using RiceXPro [50] and MSU [49], it was found that *LOC_Os09g24260*, *LOC_Os09g24200*, and *LOC_Os09g24250* had high expression levels in the ovary, embryo, and endosperm at different stages after pollination (Figure 5c,d). *LOC_Os09g24190*, *LOC_Os09g24210*, *LOC_Os09g24290*, *LOC_Os09g24320*, and *LOC_Os09g24330* had moderate expression, while other genes had very low or no expression (Figure 5c,d). Therefore, the eight genes with high and moderate expression in grain-related tissues were possible candidate genes. Among these eight genes, five have commonly significant SNPs, which are *LOC_Os09g24200*, *LOC_Os09g24250*, *LOC_Os09g24260*, *LOC_Os09g24290*, and *LOC_Os09g24320* (Table S5). They were stably detected in GWAS of both the full and *japonica* populations. Haplotype analysis of these five genes using commonly significant SNPs revealed that they were divided into two haplotypes (Hap1 and Hap2) in all germplasms. In the *japonica* subpopulation, the average TFC of Hap1 was significantly higher than that of Hap2, while in the *indica* subpopulation, all accessions belonged to Hap2 (Figure 5e; Figure S1). This result further supports the candidacy of these five genes for *qTFC9-7*. Therefore, *LOC_Os09g24200*, *LOC_Os09g24250*, *LOC_Os09g24260*, *LOC_Os09g24290*, and *LOC_Os09g24320* were candidate genes for *qTFC9-7*. Among these five genes, *LOC_Os09g24260* encodes a protein containing a WD domain and G-beta repeat domain, which is closest to the lead SNP in the full population and has more commonly significant SNPs (Table S5). *OsWD40*/*OsTTG1* also encodes a WD40 protein, and its mutations significantly reduced the accumulation of flavonoids in various organs of rice [32]. The WD40 protein is a component of the MYB-bHLH-WD40 (MBW) transcription factor complex that regulates the expression of some structural genes involved in flavonoid synthesis [54,55]. Therefore, *LOC_Os09g24260* is the most likely candidate gene in *qTFC9-7*; however, there is not enough evidence to exclude the other four genes from the list of candidates.

### 3.5. Geographic Distribution and Breeding Utilization Analysis of Favorable Haplotypes of Preferred Candidate Genes of qTFC1-6 and qTFC9-7

*LOC_Os01g59440* and *LOC_Os09g24260* are the most promising candidate genes for *qTFC1-6* and *qTFC9-7*, respectively, and they are divided into two haplotypes (Hap1 and Hap2) in all germplasms (Figure 4e and Figure 5e). *LOC_Os01g59440*^Hap1^ is the favorable haplotype of *LOC_Os01g59440* in the *japonica* subpopulation, and *LOC_Os09g24260*^Hap1^ is the favorable haplotype of *LOC_Os09g24260* in the *japonica* subpopulation. However, almost all *indica* germplasm belonged to *LOC_Os01g59440*^Hap2^ and *LOC_Os09g24260*^Hap2^.

**Figure 6:**
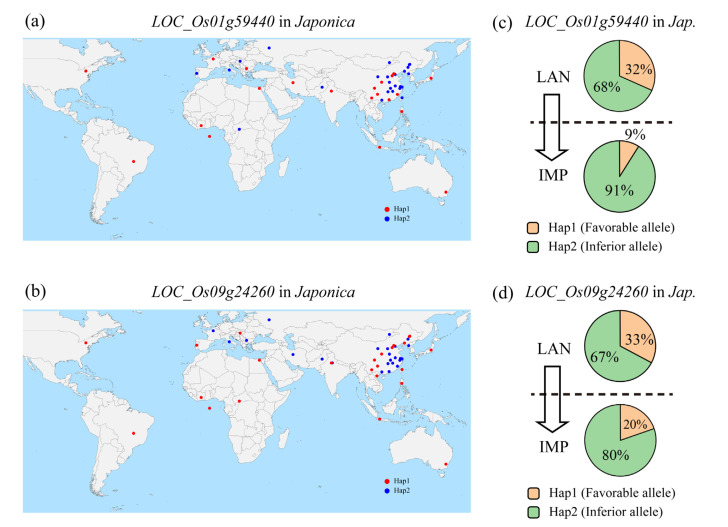
The geographic distribution and breeding utilization analysis of *LOC_Os01g59440*^Hap1^ and *LOC_Os09g24260*^Hap1^. (**a**) The geographic distribution of *LOC_Os01g59440*^Hap1^ in the *japonica* subpopulation. (**b**) The geographic distribution of *LOC_Os09g24260*^Hap1^ in the *japonica* subpopulation. (**c**) The breeding utilization of *LOC_Os01g59440*^Hap1^ in the *japonica* subpopulation. LAN represents a landrace. IMP represents improved variety. (**d**) The breeding utilization of *LOC_Os09g24260*^Hap1^ in the *japonica* subpopulation.

To investigate the geographic distribution and breeding utilization of *LOC_Os01g59440*^Hap1^ and *LOC_Os09g24260*^Hap1^ in *japonica*, we conducted geographical distribution analysis and breeding utilization analysis on them. The results showed that germplasms belonging to *LOC_Os01g59440*^Hap1^ in the *japonica* subpopulation was widely distributed across multiple regions worldwide, including areas with varying longitudes and latitudes (Figure 6a). Similarly, germplasm belonging to *LOC_Os09g24260*^Hap1^ in the *japonica* subpopulation was also widely distributed across multiple regions worldwide, including areas with varying longitudes and latitudes (Figure 6b). The proportion of *LOC_Os01g59440*^Hap1^ in landraces of the *japonica* subpopulation was 32%, while it was 9% in improved varieties of the *japonica* subpopulation (Figure 6c). This indicates that the utilization rate of *LOC_Os01g59440*^Hap1^ in improved varieties is still relatively low, and there is great potential for future applications of this favorable haplotype. Similarly, the proportion of *LOC_Os09g24260*^Hap1^ in landraces of the *japonica* subpopulation was 33%, while it was 20% in improved varieties of the *japonica* subpopulation (Figure 6d). This indicates that the utilization rate of *LOC_Os09g24260*^Hap1^ in improved varieties is also relatively low, and there is significant potential for future applications of this favorable haplotype.

## 4. Discussion

Flavonoids are known to have anti-inflammatory, antioxidative, and anticarcinogenic effects. Breeding functional rice varieties rich in flavonoids may prevent chronic diseases such as cancer and cardio-cerebrovascular disease.

In a previous study, the TFC of 164 accessions from the USDA rice mini-core collection ranged from 43.4 to 411.9 mg/100 g [53]. In our study, the TFC ranged from 90.6 to 435.9 mg/100 g. Although the range of TFC in our study did not exceed that of the previous study, we identified elite accessions with higher TFC, such as Yanshuichi (TFC = 435.91 mg/100 g), which could serve as valuable germplasm resources for breeding. The TFC in the full population showed a positively skewed distribution because the TFC of 11 accessions exceeded 300 mg/100 g, and 93% of the accessions ranged from 90 to 170 mg/100 g (Figure 1b). This suggests that a few accessions may contain favorable alleles that control flavonoid content.

We found no significant difference in TFC between the *indica* and *japonica* subpopulations (Figure 1f), and the 10 varieties with the highest TFC included three *indica* and seven *japonica* varieties, indicating no apparent relationship between TFC and geographic origin. Our findings are consistent with those of a previous study that found no significant difference in TFC between the *indica* and *japonica* subpopulations in the primary core collection from the Yunnan province of China [11]. However, Dong et al. (2014) reported significant differences between the two subpopulations for specific flavonoids [56]. For instance, two flavone C-pentosides in flag leaves, apigenin C-pentoside and luteolin C-pentoside, showed over-accumulation by 200 times in the *indica* subpopulation compared to the *japonica* subpopulation, and coumaroyl derivatives of flavone C-hexosyl-O-hexosides in flag leaves were found to have lower levels in the *indica* subpopulation compared to the *japonica* subpopulation. These results suggest that although we found no significant difference in TFC between the *indica* and *japonica* subpopulations, there may still be significant differences in specific flavonoids.

Our analysis found that among the 10 accessions with high flavonoid content, nine were landrace varieties, indicating that landraces are a promising source for identifying excellent varieties with high flavonoid content. Therefore, it is crucial to carefully collect and phenotypically identify landrace germplasm.

When looking for genes that have been reported, although some genes affecting flavonoid content were reported to be expressed in grains, no direct evidence that these genes affect flavonoid content in grains was found. Thus, they could not be regarded as genes affecting the flavonoid content of rice grains. For example, although *OsCHS24* and *OsbZIP48* are expressed in grains and leaves and affect the content of flavonoids in leaves [28,57], no evidence that *OsCHS24* and *OsbZIP48* affect the flavonoid content in grains was found. Therefore, they do not belong to the genes that affect the flavonoid content of rice grains.

When screening candidate genes, previous studies considered that if significant SNPs were located in the promoter of certain genes or belonged to non-synonymous SNPs in exons, such genes might be candidate genes [58,59], which would ignore genes with significant SNPs located in introns and 3′UTRs. However, variations in the intron or 3′UTR can also lead to changes in gene expression, thus affecting gene function [60,61,62]. To avoid disregarding potential candidate genes, we considered that genes with significant SNPs in the entire gene region, including the promoter, exon, intron, and/or 3’UTR, might be candidates. Therefore, in our study, we included *LOC_Os01g59600* and *LOC_Os01g59610* as candidate genes, even though their significant SNPs were located in the intron and 3′UTR (Figure 4e; Table S4).

The genes involved in the synthesis and regulation of flavonoids in rice have been widely studied, including structural, regulatory, transporter, and modifying enzyme genes. However, among the genes that have been reported to affect the flavonoid content in rice, there are still a few that do not belong to the known pathways of flavonoid synthesis or regulation, such as *OsRLCK160*, *POT*, and *OsCRY1b* [57,63,64], which encode a receptor-like kinase, proton-dependent oligopeptide transporter, and cryptochrome, respectively. The relationship between these proteins and flavonoid synthesis or regulatory pathways is still unknown, indicating a lack of knowledge about the mechanisms of flavonoid content in rice. Therefore, even though *LOC_Os01g59440*, *LOC_Os01g59600*, *LOC_Os01g59610*, *LOC_Os09g24200*, *LOC_Os09g24250*, *LOC_Os09g24290*, and *LOC_Os09g24320*, the seven candidate genes identified in our study for *qTFC1-6* and *qTFC9-7*, encode a brassinosteroid insensitive 1-associated receptor kinase 1 precursor, T1 family peptidas, KAZ1-Kazal-type serine protease inhibitor precursor, RAD23 DNA repair protein, agenet domain containing protein, and 2-oxo acid dehydrogenase acyltransferase domain containing protein, respectively, which appear to be unrelated to known flavonoid synthesis and regulatory pathways, they could also affect flavonoid content through other unknown pathways. For example, *LOC_Os01g59440*, encoding a brassinosteroid insensitive 1-associated receptor kinase 1 precursor, is similar to *OsRLCK160*, which encodes a receptor-like kinase that interacts with OsbZIP48 and phosphorylates it, thereby regulating the accumulation of flavonoids in rice leaves [57]. Therefore, *LOC_Os01g59440* could regulate TFC in brown rice through similar unknown mechanisms.

Another candidate gene identified in our study, *LOC_Os09g24260* of *qTFC9-7*, encodes a WD domain and G-beta repeat domain-containing protein, which is related to the known flavonoid content pathway. The WD40 protein is a component of the MYB-bHLH-WD40 (MBW) transcription factor complex that regulates the expression of some structural genes involved in flavonoid synthesis [54,55]. Therefore, *LOC_Os09g24260* is the most likely candidate gene for *qTFC9-7*.

## 5. Conclusions

In this study, we identified ten excellent rice varieties with high TFC and detected 53 QTLs associated with TFC in brown rice. From two preferred co-located QTLs, *qTFC1-6* and *qTFC9-7*, we identified eight candidate genes: *LOC_Os01g59440*, *LOC_Os01g59600*, *LOC_Os01g59610*, *LOC_Os09g24200*, *LOC_Os09g24250*, *LOC_Os09g24260*, *LOC_Os09g24290*, and *LOC_Os09g24320.* Among these genes, *LOC_Os01g59440* and *LOC_Os09g24260*, which encode a receptor-like kinase and a WD domain and G-beta repeat domain-containing protein, respectively, are the most likely candidate genes. We further analyzed the geographic distribution and breeding utilization of favorable haplotypes of these two genes. This study elucidates the genetic basis of total flavonoid content in brown rice, which could help in developing rice cultivars with high flavonoid levels that offer benefits in the prevention and adjuvant treatment of cardio-cerebrovascular diseases and cancer.

## Figures and Tables

**Table 1:** Ten rice varieties with the highest total flavonoid content.

Variety Name	Subspecies	Origin	TFC (mg/100 g)	Landrace or Improved Variety
Yanshuichi	*Ind.*	Fujian, China	435.9	landrace
Feidongtangdao	*Jap.*	Anhui, China	426.0	landrace
Lengshuigu 2	*Jap.*	Yunnan, China	407.3	landrace
Qiuqianbai	*Ind.*	Anhui, China	355.8	landrace
IRAT 669	*Jap.*	Ivory Coast	347.8	improved variety
Haobayong 1	*Jap.*	Yunnan, China	344.5	landrace
Lamujia	*Jap.*	Yunnan, China	331.5	landrace
Longhuamaohulu	*Jap.*	Hebei, China	330.6	landrace
Tianhandao	*Ind.*	Vietnam	316.4	landrace
Banjiemang	*Jap.*	Yunnan, China	313.7	landrace

**Table 2:** Information on all co-located QTLs.

QTL Name Merged	QTL Name of the Respective Populations	Chr.	Lead SNP	−log_10_(*P*)	Left SNP	Right SNP	Length of Overlapping Interval	No. of Commonly sig. SNP	Cloned Gene
*qTFC7-2*	*qTFC-Full-7-1*	7	6141196	7.91	6062744	6468059	371872	104	*Rc*
	*qTFC-Ind-7-2*	7	6069266	15.76	5921983	6434616			*Rc*
*qTFC1-6*	*qTFC-Full-1-5*	1	34468941	8.58	34464710	34491418	26708	36	
	*qTFC-Jap-1-4*	1	34467581	8.81	34464710	34491418			
*qTFC9-7*	*qTFC-Full-9-5*	9	14419332	8.29	14014590	14471720	348111	30	
	*qTFC-Jap-9-2*	9	14210477	7.66	14111737	14459848			
*qTFC1-2*	*qTFC-Full-1-1*	1	6313712	5.75	6251537	6340212	88675	21	
	*qTFC-Jap-1-2*	1	6368734	8.65	6226226	6461894			
*qTFC4-3*	*qTFC-Full-4-1*	4	25197717	6.79	25029953	25399507	369554	11	
	*qTFC-Ind-4-2*	4	25197717	6.50	24981902	25399507			
*qTFC7-3*	*qTFC-Full-7-2*	7	6749334	6.18	6749334	6953995	129030	7	
	*qTFC-Ind-7-3*	7	6782095	7.96	6723681	6878364			
*qTFC5-4*	*qTFC-Full-5-1*	5	22309638	8.23	22280681	22468262	47736	2	
	*qTFC-Jap-5-1*	5	22317452	5.61	22309638	22357374			
*qTFC6-1*	*qTFC-Full-6-1*	6	2046161	6.35	2046147	2046165	18	4	
	*qTFC-Ind-6-1*	6	2046161	6.25	2046147	2046688			
*qTFC1-4*	*qTFC-Full-1-3*	1	25460333	6.54	25454033	25496676	16256	0	
	*qTFC-Jap-1-3*	1	25479651	5.11	25478991	25495247			
*qTFC3-3*	*qTFC-Jap-3-1*	3	10736942	5.67	10736930	11139080	155123	0	
	*qTFC-Ind-3-2*	3	11092283	5.66	10983957	11271479			
*qTFC8-3*	*qTFC-Full-8-1*	8	20869072	5.63	20844005	20939712	95707	0	
	*qTFC-Ind-8-1*	8	20653491	9.11	20648615	20942578			
*qTFC10-2*	*qTFC-Full-10-1*	10	11225165	7.48	11220698	11225165	4467	0	
	*qTFC-Jap-10-1*	10	11228250	6.96	11137616	11704622			
*qTFC11-4*	*qTFC-Full-11-1*	11	23285861	5.44	23111961	23406804	114658	0	
	*qTFC-Jap-11-2*	11	23170730	5.56	23170584	23285242			

## Data Availability

All data in the present study are available in the public database, as mentioned in Section 4.

## Supplementary Materials

The following supporting information can be downloaded at: https://www.mdpi.com/article/10.3390/genes14091684/s1. xxx. Figure S1. Haplotype analysis of the other four candidate genes of *qTFC9-7*. Table S1. The information of 633 cultivated rice accessions. Table S2. Information of the QTLs associated with total flavonoid content in brown rice identified by GWAS in the full, *indica*, and *japonica* populations. Table S3. Significant SNPs of *Rc* in the full and *indica* populations. Table S4. All significant SNPs from the full and *indica* populations within the LD interval of *qTFC1-6*. Table S5. All significant SNPs from the full and *japonica* populations within the LD interval of *qTFC9-7*.
