# Supplementary material for: Genome-Wide Association Study Reveals the Genetic Basis of Total Flavonoid Content in Brown Rice
Source: Genes (Basel). 2023 Aug 25;14(9):1684. doi: 10.3390/genes14091684 (PMC10531027; doi:10.3390/genes14091684)
Supplement: Supplementary file 1 [file genes-14-01684-s001.zip › supplymentary figure S1.pdf]

LOC_Os09g24200						
Hap.	Promoter	Intron	*Ind.*		*Jap.*	
	-617	1579	Num.	GABA	Num.	GABA
Hap1	G	T	0	—	46	158.80[a]
Hap2	A	C	319	135.35	175	128.36[b]
$-\log_{10}(P)$ Full	4.3	4.1				
$-\log_{10}(P)$ *Jap.*	4.1	3.9				

LOC_Os09g24250																	
	Promoter							Exon		Intron	3'UTR				*Ind.*		*Jap.*	
	-1527	-588	-570	-569	-190	-189	-185	406	432	1217	4857	4861	4868	4881	Num.	GABA	Num.	GABA
Hap1	T	G	T	A	T	A	T	A	A	A	A	G	T	G	0	—	36	169.01[a]
Hap2	C	A	C	G	C	C	G	G	G	C	G	A	C	A	348	135.85	174	128.37[b]
$-\log_{10}(P)$ Full	4.5	5.3	5.1	5.1	4.2	4.1	4.1	4.3	4.5	4.6	5.9	6.3	6.5	7.2				
$-\log_{10}(P)$ *Jap.*	4.0	5.3	5.1	5.1	4.0	3.8	3.8	3.8	3.8	3.8	5.7	5.7	6.2	6.2				

LOC_Os09g24290																				
Hap.	Promoter				Intron		Exon										*Ind.*		*Jap.*	
	-1490	-1481	-1101	-219	3074	3092	3297	3334	3364	3633	3737	3741	3782	3785	5576	5613	Num.	GABA	Num.	GABA
Hap1	A	T	C	A	T	C	A	C	C	G	A	A	T	T	G	T	0	—	41	161.24[a]
Hap2	C	C	T	G	C	T	G	T	T	A	G	G	C	C	A	C	357	135.75	178	128.37[b]
$-\log_{10}(P)$ Full	4.4	4.6	4.4	4.4	4.2	4.1	5.1	5.4	4.7	4.5	6.0	6.1	5.0	4.9	4.8	4.3				
$-\log_{10}(P)$ *Jap.*	4.0	4.3	4.2	3.8	3.8	3.7	4.7	4.7	4.0	3.8	5.1	5.2	4.0	3.9	4.3	4.1				

LOC_Os09g24320												
Hap.	Promoter								*Ind.*		*Jap.*	
	-856	-1137	-1151	-1178	-1181	-1219	-1715	-1760	Num.	GABA	Num.	GABA
Hap1	T	T	G	T	C	G	T	T	0	—	36	169.01[a]
Hap2	G	C	A	C	T	A	G	G	348	135.85	174	128.37[b]
$-\log_{10}(P)$ Full	5.6	5.3	6.0	4.2	4.1	6.4	4.6	4.2				
$-\log_{10}(P)$ *Jap.*	4.8	3.7	4.0	4.1	3.8	5.8	4.1	4.1				

Supplementary figure S1. Haplotype analysis of the other four candidate genes of *qTFC9-7*.
